# Sperm morphological abnormalities in autosomal dominant polycystic kidney disease are associated with the Hippo signaling pathway via PC1

**DOI:** 10.3389/fendo.2023.1130536

**Published:** 2023-04-19

**Authors:** Wei-Hui Shi, Zhi-Yang Zhou, Mu-Jin Ye, Ning-Xin Qin, Zi-Ru Jiang, Xuan-You Zhou, Nai-Xin Xu, Xian-Lin Cao, Song-Chang Chen, He-Feng Huang, Chen-Ming Xu

**Affiliations:** ^1^Obstetrics and Gynecology Hospital, Institute of Reproduction and Development, Fudan University, Shanghai, China; ^2^International Peace Maternity and Child Health Hospital, Shanghai Jiao Tong University School of Medicine, Shanghai, China; ^3^Department of Assisted Reproductive Medicine, Shanghai First Maternity and Infant Hospital, Tongji University School of Medicine, Shanghai, China; ^4^Research Units of Embryo Original Diseases, Chinese Academy of Medical Sciences (No. 2019RU056), Shanghai, China

**Keywords:** autosomal dominant polycystic kidney disease, *PKD1*, male infertility, sperm flagella, the Hippo pathway

## Abstract

**Background:**

Autosomal dominant polycystic kidney disease (ADPKD) is a hereditary kidney disorder mostly caused by mutations in *PKD1* or *PKD2* genes. Here, we report thirteen ADPKD males with infertility and investigated the sperm morphological defects associated with PC1 disruption.

**Methods:**

Targeted next-generation sequencing was performed to detect *PKD1* variants in patients. Sperm morphology was observed by immunostaining and transmission electron microscopy, and the sperm motility was assessed using the computer-assisted sperm analysis system. The Hippo signaling pathway was analyzed with by quantitative reverse transcription polymerase chain reaction (qPCR) and western blotting *in vitro*.

**Results:**

The ADPKD patients were infertile and their sperm tails showed morphological abnormalities, including coiled flagella, absent central microtubules, and irregular peripheral doublets. In addition, the length of sperm flagella was shorter in patients than in controls of in in. *In vitro*, ciliogenesis was impaired in *Pkd1*-depleted mouse kidney tubule cells. The absence of PC1 resulted in a reduction of MST1 and LATS1, leading to nuclear accumulation of YAP/TAZ and consequently increased transcription of *Aurka*. which might promote HDAC6-mediated ciliary disassembly.

**Conclusion:**

Our results suggest the dysregulated Hippo signaling significantly contributes to ciliary abnormalities in and may be associated with flagellar defects in spermatozoa from ADPKD patients.

## 1 Introduction

Autosomal dominant polycystic kidney disease (ADPKD) is one of the most common monogenic kidney disorders with an estimated prevalence of one in 1000 live births. ADPKD is characterized by bilateral renal cysts and extra-renal manifestations, including aortic aneurysm, intracranial aneurysm and cysts in the liver, pancreas, seminal vesicles, epididymides and testes (1, 2). Mutations in *PKD1* (OMIM 601313) and *PKD2* (OMIM 173910) are the most common causes of ADPKD, which account for 80-85% and 10-15% of cases, respectively (3). Polycystin-1 (PC1), a 4303 amino acid product of *PKD1* gene, acts as a transmembrane glycoprotein with a large extracellular amino terminus, 11 membrane-spanning domains and a short intracellular carboxy terminus. Polycystin-2 (PC2), encoded by *PKD2*, functions as a non-selective cation channel permeable for to calcium ions, which co-localizes with PC1 at the primary cilia of the renal epithelia and plays a vital role in mechanosensation (4, 5).

Since the first case of a 32-year-old man with ADPKD suffering from infertility and seminal vesicle cysts was reported in 1995, male infertility in ADPKD has gradually gained attention (6–10). It has recently been reported that seminal megavesicles are found in 31% of patients with *PKD1* mutations and may be associated with the male infertility observed in ADPKD patients (11, 12). Primary cilia in the kidney are microtubule-based organelles that protrude from the surface of epithelial cells. Disruption of PC1 or PC2 causes defects in primary cilia, leading to the development of ADPKD. Similar to primary cilia, the sperm flagellum is a ciliary organelle with nine peripheral microtubule doublets and two central microtubule singlets (9 + 2 axoneme) that whip back and forth to propel the sperm, which is essential for male fertility. *Pkd2* has been reported to be highly expressed in mature sperms of Drosophila (13) and PC1 has also been observed in on human sperm proteomics studies (14). Moreover, Okada et al. discovered that four infertile men with immotile spermatozoa and abnormal flagellar ultrastructure were all diagnosed with ADPKD, suggesting that PC1 and PC2 may play a vital role in the structure of sperm flagella.

The Hippo signaling pathway, initially identified by genetic mosaic screens for suppressor genes associated with tissue overgrowth in Drosophila, regulates cell proliferation, death and differentiation to maintain organ size and tissue homeostasis (15). The components of this pathway are highly conserved in mammals and consist of MST1/2, LATS1/2, SAV1, MOB1, and YAP/TAZ (16). When upstream stimuli, such as mechanotransduction, cell polarity, and G-protein-coupled receptor signals, trigger the of the Hippo pathway kinase cascade, the kinase activity of MST1/2 which phosphorylates SAV1, LATS1/2, and MOB1, is enhanced (17, 18). Activated LATS1/2, accelerated by interaction with MOB1, directly phosphorylates the transcriptional co-activators YAP/TAZ, leading to their sequestration in the cytoplasm. It has recently been reported that depletion of *Yap* or *Taz* leads to ciliary abnormalities in zebrafish and renal cysts in mice respectively, suggesting that the Hippo signaling pathway may promote ciliogenesis and cyst formation (19, 20).

In this study, we identified thirteen ADPKD patients with mutations in *PKD1* using targeted next-generation sequencing (NGS) and observed poor semen quality and abnormal sperm morphology. *In vitro*, we found that the defect of *Pkd1* resulted in decreased in MST1 and LATS1, which promoted ciliary disassembly via the AURKA/HDAC6 complex. Our results suggest that suppressed Hippo signaling in lead to a boost in ciliary disassembly, which may also be a mechanism of impaired sperm flagella in ADPKD.

## 2 Methods

### 2.1 Subjects

All the subjects of in this study were recruited from the reproductive outpatient department of the International Peace Maternity and Child Health Hospital (IPMCH), Shanghai Jiao Tong University School of Medicine from January 2018 to September 2022. Genomic DNA of was extracted from peripheral blood samples of all subjects. A gene panel related to polycystic kidney diseases (PKD), containing *VHL*, *TSC1*, *TSC2*, *UMOD*, *PKD1*, *PKD2*, *MUC1*, and *PKHD1* genes, was detected by NGS as previously described (21). Detected variants were confirmed with by Sanger sequencing and interpreted and classified according to the ACMG guideline (22).

### 2.2 Semen analysis

Sperm samples from all subjects were collected by masturbation after 3 days of sexual abstinence. After liquefaction, sperm concentration and motility were assessed using the computer-assisted sperm analysis (CASA) system according to the World Health Organization laboratory manual (23). Semen smears were prepared by spreading the sperm suspension on a microscope the slide for subsequent sperm immunofluorescence staining.

### 2.3 Transmission electron microscopy (TEM)

Semen samples were centrifuged at 800 g for 15 minutes and washed three times with 0.01M phosphate buffered saline (PBS, pH 7.4). After fixation with pre- cooled 2.5% glutaraldehyde (in 0.1 M PBS buffer) for two hours, samples were washed twice in PBS (10 minutes each time) and post-fixed with osmium tetroxide for two hours at 4°C. Samples were dehydrated with in cold 30%, 50%, 70%, 80%, 95% and 100% ethanol for 10 minutes (100% ethanol repeated once). After being embedded with resin, the samples were cut with into thin sections and stained with lead citrate. Images were captured with a PHILIP CM-120 transmission electron microscope.

### 2.4 Cell culture

*Pkd1*-depleted mouse kidney tubule cell lines (*Pkd1*^+/-^ and *Pkd1*^-/-^ cells) (a kind gift from Dr. Changlin Mei of Changzheng Hospital, Second Military Medical University, Shanghai, China) were cultured in Dulbecco’s modified Eagle’s medium/Ham’s F-12 medium (DMEM/F12) (Gibco) supplemented with 2% fetal bovine serum (Gibco), Insulin-Transferrin-Selenium (41400045, Gibco), triiodothyronine (2 x 10^-9^ M, T5516, Sigma) and recombinant γ-interferon (10 units/ml, Sigma) at 33°C. For cilia formation, cells were transferred to 37°C and grown in γ-interferon-free medium for 7 days.

### 2.5 Cell growth detection

Cells were cultured with in equal amounts (1 x 10^4^ per well) and counted daily for 7 days using a hemocytometer. Experiments were carried out in triplicate.

### 2.6 Bromodeoxyuridine (BrdU) cell proliferation assay

Cells were grown on the coverslips in a 24-well plate to 70-90% confluence. BrdU (10μM) was added for two hours at 33°C. The cells were then washed three times with 0.01 M PBS and fixed with 4% paraformaldehyde. After washing three times again with PBS, the cells were incubated in 2 mol/L HCL for 30 minutes and then neutralized with 0.1 M sodium borate buffer three times for 15 minutes each. BrdU staining was followed by standard immunofluorescence protocols as described below.

### 2.7 Real-time quantitative reverse transcription polymerase chain reaction (qPCR)

Total RNA was isolated with RNAiso Plus (No.9108Q, Takara, Japan) and reversed to cDNA with using a PrimeScript™ RT reagent Kit with gDNA Eraser (No. RR047Q, Takara, Japan) according to the manufacturer’s instructions. Quantitative PCR (qPCR) was performed with using TB Green™ Premix Ex Taq™ (Tli RNaseH Plus) (No. RR420Q, Takara, Japan).

### 2.8 Western blotting

For western blotting analysis, cells and sperms were lysed on ice with RIPA buffer (No. P0013B; Beyotime, Shanghai, China) supplemented with InStabTM Phosphatase Inhibitor Cocktail (No.20109ES05, Yeasen, Shanghai, China) and InStabTM Protease Cocktail (No.20124ES03, Yeasen) for 30 minutes and cleared by centrifugation at 4°C, 12,000 rpm for 10 minutes. Protein concentrations were quantified by Pierce™ BCA Protein Assay Kit (No.23225, Thermo Fisher, USA). After denaturation, total protein was fractionated by SDS-polyacrylamide gel electrophoresis and transferred to polyvinylidene difluoride (PVDF) membranes. Membranes were blocked with 5% non-fat milk in TBST buffer for one hour and incubated with primary antibodies overnight at 4°C. Horseradish peroxide- conjugated secondary antibodies (Cell Signaling Technology, 1:1000) were incubated for one hour at room temperature and blots were visualized by ECL Chemiluminescent Substrate Kit (No.36222ES76, Yeasen). Primary antibodies used in for western blotting included acetylated α-tubulin (T6973, Sigma, 1:2000), α-tubulin (11224-1-AP, Proteintech, 1:1000), phospho-YAP (4911, Cell Signaling Technology, 1:1000), YAP (4912, Cell Signaling Technology, 1:1000), TAZ (4883, Cell Signaling Technology, 1:1000), MST1 (3682, Cell Signaling Technology, 1:1000), MST2 (3952, Cell Signaling Technology, 1:1000), MOB1 (13730, Cell Signaling Technology, 1:1000), phospho-MOB1 (8699, Cell Signaling Technology, 1:1000), SAV1 (13301, Cell Signaling Technology, 1:1000), LATS1 (3477, Cell Signaling Technology, 1:1000), AURKA (610938, BD Biosciences, 1:1000), PRM1 (HPA055150, Sigma, 1:500), and GAPDH (2118, Cell Signaling Technology, 1:1000).

### 2.9 Immunofluorescence staining

Cells grown on coverslips or spermatozoa on semen smears were fixed with 4% paraformaldehyde for 15 minutes at room temperature. After the fixation, samples were washed three times in 1 x PBS for 5 minutes each and then blocked in 1 x PBS with 0.3% Triton™ X-100 and 5% normal serum for one hour. Primary antibodies were incubated overnight at 4°C. Alexa Fluor 488 or 594 conjugated secondary antibodies (Invitrogen) were incubated at for one hour at room temperature. Cell nuclei were stained with DAPI (Vector labs H-1200). Images were captured using an the SP8 confocal microscope (Leica Microsystems, Wetzlar, Germany). Primary antibodies used for immunofluorescence staining included acetylated a-tubulin (T7451, Sigma, 1:250), ZO-1 (339100, Invitrogen, 1:100), YAP/TAZ (8418, Cell Signaling Technology, 1:150).

### 2.10 Data analysis

The length of cell cilia or sperm flagella and protein bands from western blotting were measured or quantified with using Image J. T wo-tailed *P* values were calculated with unpaired Student’s *t*-test. The significance level of was set at 0.05. Graphs were generated with GraphPad Prism software.

## 3 Results

### 3.1 Genetic diagnosis and semen analysis of thirteen ADPKD patients

In our reproductive genetics department, thirteen patients with renal cysts or a family history of cystic kidney disease were highly suspected of having polycystic kidney disease. Targeted NGS was performed to detect variants associated with the symptoms. After variant calling and annotation, nine pathogenic and four likely pathogenic *PKD1* variants were identified in these patients (**Table 1**). In particular, one patient (No. 8) harbored two variants that were classified as likely pathogenic and variants of uncertain significance (VUS) according to the ACMG-AMP guidelines (22).

**Table 1:** Genetic information of the ADPKD patients.

Patients	Gene	Exon/Intron	Nucleotide change	Amino acid change	Zygosity	De novo/Inherited	Variant Classification
1	*PKD1*	EX18	c.7288C>T	p. Arg2430Ter	heterogeneous	inherit	pathogenic
2	*PKD1*	EX14	c.6465_6466delGC	p. Leu2155fs18Ter	heterogeneous	inherit	pathogenic
3	*PKD1*	EX15	c.3670G>T	p. Glu1224Ter	heterogeneous	*de novo*	pathogenic
4	*PKD1*	EX5	c.937G>T	p. Glu313Ter	heterogeneous	inherit	pathogenic
5	*PKD1*	EX46	c.12448C>T	p. Arg4150Cys	heterogeneous	inherit	likely pathogenic
6	*PKD1*	EX28	c.9578C>T	p. Pro3193Leu	heterogeneous	inherit	likely pathogenic
7	*PKD1*	EX10	c.1987C>T	p. Gln663Ter	heterogeneous	inherit	pathogenic
8	*PKD1*	EX3	c.350T>C	p. Leu117Ser	heterogeneous	*de novo*	likely pathogenic
	*PKD1*	EX46	c.12455A>C	p. Lys4152Thr	heterogeneous	inherit	VUS
9	*PKD1*	EX13	c.3067C>T	p. Gln1023Ter	heterogeneous	inherit	pathogenic
10	*PKD1*	EX45	c.12366G>A	p. Trp4122Ter	heterogeneous	inherit	pathogenic
11	*PKD1*	EX46	c.12712C>T	p. Gln4238Ter	heterogeneous	NA	pathogenic
12	*PKD1*	EX15	c.4997G>A	p. Trp1666Ter	heterogeneous	inherit	pathogenic
13	*PKD1*	EX15	c.6890A>C	p. His2297Pro	heterogeneous	NA	likely pathogenic

NA, not available; VUS, Variant of uncertain significance.

These patients all suffered from male infertility. Two patients (Nos. 8 and 11 in **Table 1**) had severe oligospermia and three patients (Nos. 9, 10 and 12 in **Table 1**) had azoospermia. Semen samples from the other eight patients and sixteen age-matched controls were analyzed by CASA (**Table 2**). Compared to controls, the percentage of sperm with progressive motility (PR, %) and sperm with normal morphology in ADPKD patients was apparently less (*P* < 0.001) and below the reference limit (**Table 2**). In addition, the parameters of sperm curvilinear velocity (VCL), straight-line velocity (VSL), average path velocity (VAP), and amplitude of lateral head displacement (ALH) were also significantly lower in ADPKD patients than that in healthy controls (*P* < 0.05).

**Table 2:** Sperm characteristics of ADPKD patients and age-matched healthy controls.

Parameters	Male controls (n=16)	ADPKD patients (n=8)	*P* value
Age	37.56 ± 5.39	37.50 ± 6.16	0.980
Semen *v*olume (ml)	3.25 ± 1.41	2.41 ± 1.29	0.173
Sperm *c*oncentration (10^6^ *p*er *m*l)	60.36 ± 30.27	66.96 ± 61.19	0.724
Normal *f*orms (%)	5.63 ± 1.41	2.75 ± 1.91	<0.001
Progressive *m*otility (PR, %)	50.77 ± 10.59	17.23 ± 13.17	<0.001
Non-progressive *m*otility (NP, %)	7.15 ± 4.44	13.22 ± 16.03	0.325
Immotile *s*permatozoa (IM, %)	42.81 ± 10.63	70.60 ± 15.62	<0.001
Curvilinear *v*elocity (VCL, μm/s)	101.15 ± 23.90	56.09 ± 24.04	<0.001
Straight-line *v*elocity (VSL, μm/s)	46.72 ± 5.85	28.37 ± 11.44	<0.001
Average *p*ath *v*elocity (VAP, μm/s)	60.36 ± 8.88	35.61 ± 14.23	<0.001
Linearity (LIN, %)	49.63 ± 8.89	56.10 ± 9.35	0.113
Amplitude of *l*ateral *h*ead *d*isplacement (ALH, μm)	5.34 ± 1.56	3.38 ± 1.38	0.006
Straightness (STR, %)	77.71 ± 7.82	80.90 ± 7.69	0.354
Beat-cross *f*requency (BCF, Hz)	23.74 ± 2.72	22.62 ± 4.04	0.426
Wobble (WOB, %)	61.87 ± 5.45	67.45 ± 6.18	0.034

### 3.2 Morphological defects of spermatozoa in ADPKD patients

**Figure 1:**
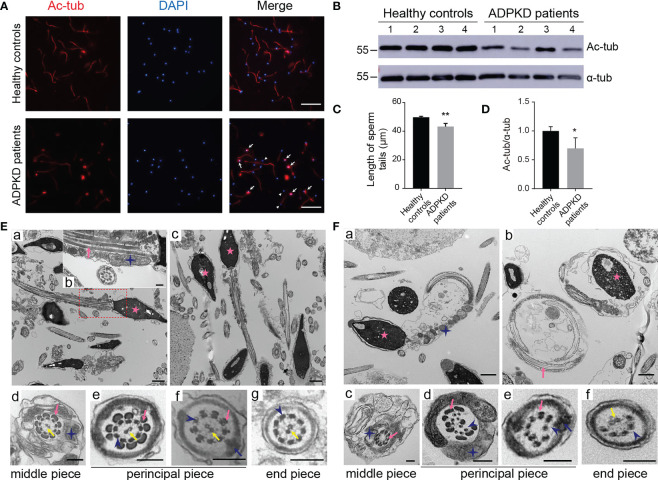
Morphology of sperm from healthy controls and ADPKD patients. **(A)** Immunofluorescence staining of sperms. White arrows indicate curled sperm flagella stained with acetylated α-tubulin (red signal) in ADPKD patients. White arrowheads indicate small sperm heads stained with 4’,6-diamidino-2-phenylindole (DAPI) (blue signal). Scale bar: 50μm. **(B)** Representative western blotting images of acetylated α-tubulin and α-tubulin in sperms from healthy controls and ADPKD patients (n=4). **(C)** Comparison of sperm tail length between controls and ADPKD patients (n=4). At least 30 sperms were counted from each individual observed from three random fields per slide. **(D)** Relative intensities of acetylated α-tubulin/α-tubulin in sperms. **(E)** Ultrastructure of sperm flagella from healthy controls. (a, c) Longitudinal sections of normal spermatozoa with straight sperm tails. (b) Magnification of the dotted area in (a). (d-g) Cross-sections of spermatozoa at various levels with regular arrangement of axonemes. **(F)** Ultrastructure of sperm flagella from ADPKD patients. (a, b) Sperm flagella in ADPKD patients are coiled and wrapped around the heads. (c-f) Disruption of axonemes or outer dense fibers in the cross-section of sperm tails. Blue stars indicate mitochondria, pink arrows indicate outer dense fibers, blue arrows indicate fibrous sheath, yellow arrows indicate central axoneme; blue arrowheads indicate peripheral axonemes, and pink stars indicate nuclei. Scale bars: Ea, Ec, Fa, Fb: 1μm; Eb, Ed-g, Fc-f: 200nm. Error bars indicate standard deviation of three independent experiments (*P < 0.05, **P < 0.01; Student’s t test).

To further investigate the sperm abnormalities in ADPKD patients, we observed sperm morphology by acetylated α-tubulin immunostaining, a protein specific for flagellar microtubules. In *PKD1*-mutant patients, spermatozoa showed a high rate of coiled flagella and some had small heads (**Figure 1A**). Moreover, the length of acetylated α-tubulin staining on sperm flagella of was shorter in patients than that of in control s and the expression of acetylated α-tubulin was also lower in ADPKD patients (**Figures 1B–D**).

Detected by TEM, the normal ultrastructure of sperm flagella was shown in controls, including nine microtubular doublets and two central singlets along the entire tail, the mitochondrial sheath and outer dense fibers (ODFs) in the middle piece, as well as the fibrous sheath in the principal piece (**Figures 1E**a-g). However, longitudinal sections of sperm from ADPKD patients showed coiled flagella wrapped around the heads forming a loop (**Figures 1F**a, b). Additionally, the intrinsic structure of the axonemes was dramatically disrupted in of. In transverse sections, the absence of central singlets and irregular arrangements or a decrease in the number of doublet microtubules and ODFs were frequently observed in different pieces of sperm flagella (**Figures 1F**c-f).

### 3.3 Compromised ciliogenesis in *Pkd1*-depleted mouse kidney tubule cells

**Figure 2:**
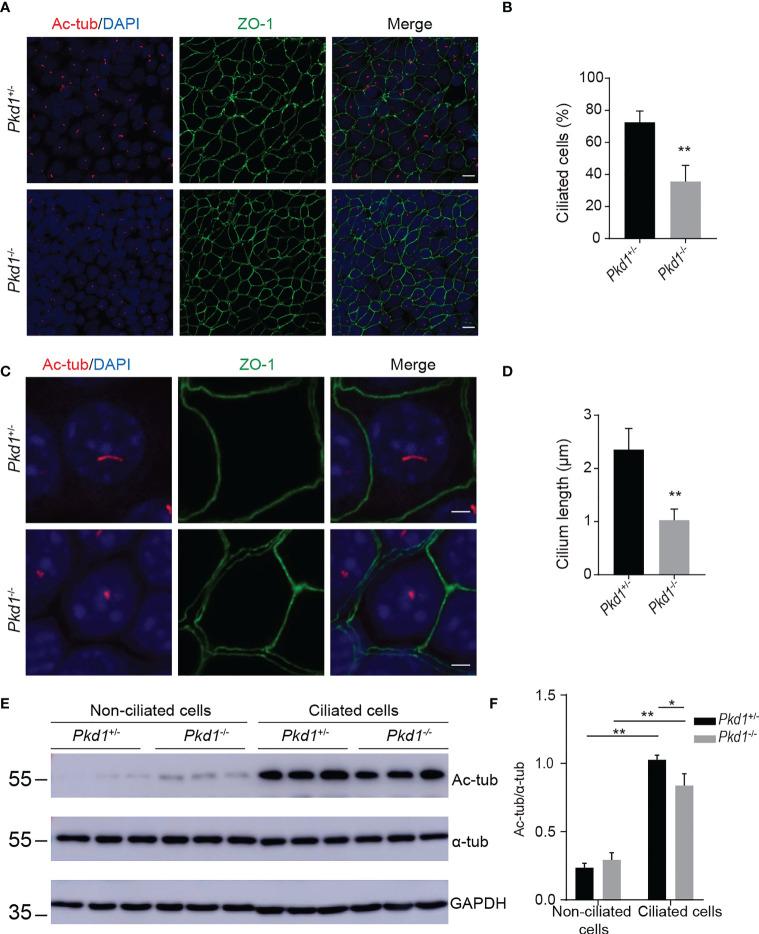
Compromised ciliary integrity in *Pkd1*-depleted mouse kidney tubule cells. **(A)** Immunofluorescence images of homozygous or heterozygous *Pkd1-* knockout cells. Cilia and tight junctions were stained with acetylated α-tubulin (red) and ZO-1 (green), respectively. Nuclei were stained with DAPI (blue). Scale bar: 25μm. **(B)** Percentage of ciliated cells in *Pkd1^+/-^* or *Pkd1^-/-^* cells. At least 200 cells were counted from three random fields per slide. **(C)** Ciliary morphology in *Pkd1^+/-^* or *Pkd1^-/-^* cells. Scale bar: 2.5μm. **(D)** Quantifications of cilia length in *Pkd1^+/-^* or *Pkd1^-/-^* cells. At least 50 cells were counted from three random fields per slide. **(E)** Representative western blotting images of acetylated α-tubulin (Ac-tub) and α-tubulin (α-tub) with GAPDH loading control in non-ciliated and ciliated *Pkd1*-depleted mouse kidney tubule cells. **(F)** Relative intensities of acetylated α-tubulin/α-tubulin in cells. Error bars indicate standard deviation of three independent experiments (*P < 0.05, **P < 0.01; Student’s *t* test).

PC1, encoded by *PKD1*, is one of the structural components of primary cilia. To evaluate the ciliogenesis of *Pkd1*-depleted mouse kidney tubule cells, the cells were grown at 37°C for 7 days without interferon-γ. After induction, the number of ciliated cells labeled with acetylated α-tubulin of in homozygous *Pkd1*-depleted (*Pkd1*^-/-^) cells with less than that in heterozygous *Pkd1*-depleted (*Pkd1*^+/-^) cells (**Figures 2A, B**). Furthermore, the length of cilia of in *Pkd1*^-/-^ cells was significantly shorter than that in *Pkd1*^+/-^ cells (**Figures 2C, D**). In addition, the acetylation level of α-tubulin in *Pkd1*^-/-^ cells was lower compared to *Pkd1*^+/-^ cells after ciliogenesis, whereas there was no significant difference between *Pkd1*^-/-^ and *Pkd1*^+/-^ cells before ciliogenesis (**Figures 2E, F**). These results suggest an important role of *PKD1* in ciliogenesis, the underlying mechanism of which may be similar to the defects in sperm flagella in ADPKD.

### 3.4 Increased nuclear accumulation of YAP/TAZ in the absence of PC1

**Figure 3:**
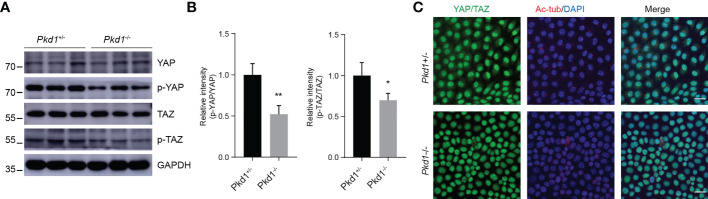
Increased nuclear accumulation of YAP/TAZ in the absence of PC1. **(A)** Representative western blotting images of YAP, phospho-YAP, TAZ and phospho-TAZ in ciliated *Pkd1^+/-^* and *Pkd1^-/-^* cells. **(B)** Relative intensities of phospho-Yap to Yap and phospho-TAZ to TAZ. Error bars indicates standard deviation of three independent experiments (*P < 0.05, **P < 0.01; Student’s *t* test). **(C)** Immunofluorescence staining of YAP/TAZ (green), acetylated α-tubulin (red), and DAPI (blue). Scale bar: 25μm.

Several studies on in animal models have shown that YAP/TAZ, known as transcriptional coactivators that promote cell proliferation, are critical for ciliogenesis and kidney development (16, 17). Since YAP/TAZ may be involved in the pathogenesis of ADPKD, we detected the expression of YAP and TAZ in *Pkd1*-depleted kidney tubule cells. It was shown that the expression patterns of total YAP/TAZ were qualitatively parallel, with no difference between *Pkd1*^+/-^ cells and *Pkd1*^-/-^ cells (**Figures 3A, B**). Nevertheless, the both phospho-YAP and phospho-TAZ were reduced in homozygous cells. Moreover, YAP/TAZ were prone to accumulate in the nuclei in of *Pkd1*^-/-^ cells (**Figure 3C**), which would enhance their growth-promoting function. Indeed, *Pkd1*^-/-^ cells were found to proliferate faster than *Pkd1*^+/-^ cells detected by the cell growth curve and BrdU assay (**Figures S1A–C**).

### 3.5 Disruption of PC1 restrained the Hippo signaling pathway

**Figure 4:**
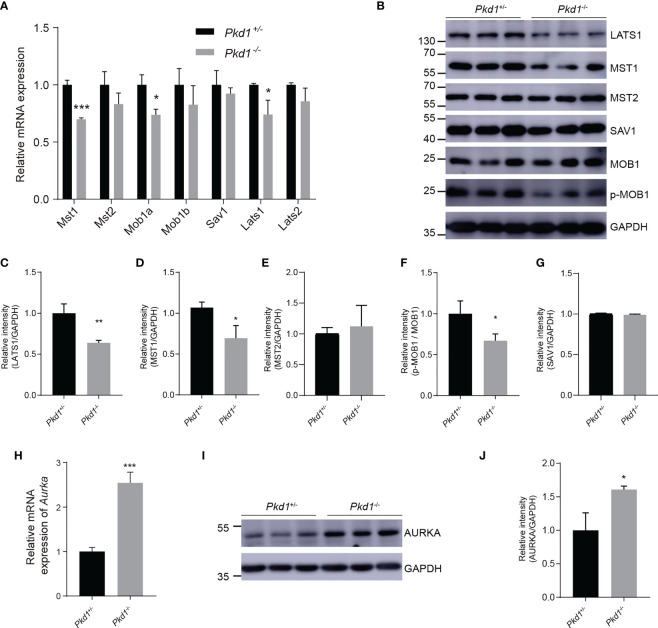
The restrained Hippo signaling pathway and increased AURKA. **(A)** Relative mRNA expression of core components of the Hippo kinase cascade. **(B)** Representative western blotting images of core components of the Hippo signaling pathway with GAPDH as the loading control. **(C–G)** Relative intensities of LATS1, MST1, MST2, p-MOB1, and SAV1, respectively. **(H)** Relative mRNA expression of *Aurka* in *Pkd1^+/-^* and *Pkd1^-/-^* cells. **(I)** Representative western blotting images of AURKA. **(J)** Relative intensity of AURKA. All error bars indicate standard deviation of three independent experiments (*P < 0.05, **P < 0.01, ***P < 0.001; Student’s *t* test).

The activation of YAP/TAZ is mainly regulated by the Hippo kinase cascade. To further explore the cause of reduced phospho-YAP/TAZ, we detected core components in of the Hippo signaling pathway. It was found that both MST1 and LATS1 were reduced in *Pkd1*^-/-^ cells (**Figures 4A–E**), with a significant difference compared with to in *Pkd1*^+/-^ cells. Moreover, phospho-MOB1, which is regulated by MST1, appeared to be reduced in *Pkd1*^-/-^ cells in concordance with the reduction of MST1 (**Figure 4F**). The scaffold protein SAV1, which is also phosphorylated by MST1 and complexes with MST1 to activate LATS1/2 and MOB1, was not significantly different between the two cell types (**Figure 4G**). Activated YAP/TAZ drives the transcription factor TEAD4 to bind to the promoter region of the Aurora A kinase (*Aurka*), which has been demonstrated to promote ciliary disassembly by activating the histone deacetylase 6 (HDAC6) (24). We found a significantly increased expression of AURKA in the absence of PC1 (**Figures 4H–J**), suggesting that the enhanced ciliary disassembly contributes to the compromised ciliary integrity in *Pkd1*-depleted cells.

## 4 Discussion

The sperm tail is the propulsion system, so the abnormal structures of the flagella are responsible for sperm immobility, which is also a typical feature of male infertility (25). Accumulating evidence suggests that sperm motility is associated with pathologies of the sperm tail, including defects in the mitochondrial sheath, the outer dense fiber, the fibrous sheath or the axoneme (26). For instance, SPAG6 is a scaffold protein that localized in the central microtubules of flagellar axonemes. The male *Spag6* knockout mice were infertile and characterized by abnormal sperm flagella, such as the loss of central microtubules, disorganized ODFs and disrupted fiber sheaths (27).In a study of 247 patients with asthenospermia, flagellar abnormalities were frequently detected (28). In this study, we found that men with ADPKD, an inherited cystic kidney disease, were associated with male infertility. The spermatozoa of these patients had obvious flagellar defects, such as coiled and short flagella, missing central microtubules, and irregular peripheral doublets. Primary ciliary dyskinesia (PCD), another ciliopathy, was also associated with male infertility. In an adult cohort of PCD, 37 males out of 49 (75.5%) PCD patients were infertile (29). Thus, the sterile manifestations of ciliopathies may be due to a similar mechanism of ciliogenesis and flagellogenesis.

The core of the sperm flagellum is the axoneme, which consists of a central pair of singlet microtubules and nine surrounding microtubular doublets (30). Two major components of microtubules, α-tubulin and β-tubulin, undergo various post-translational modifications (PTMs), including acetylation, tyrosination, glutamylation, and glycation (31). As the most common PTM, acetylation of α-tubulin, first identified in axonemes of Chlamydomonas at the of lysine 40 (32), is a marker of stable microtubules, such as cilia and flagella. Acetylated α-tubulin is involved in many cellular processes, including cilium assembly, cellular signal and intracellular transport (33, 34). In semen samples of from ADPKD patients, we observed that the acetylation level of α-tubulin was significantly reduced. Intriguingly, a similar downward trend in the ratio of sperm acetylated α-tubulin/α-tubulin has been reported in individuals with asthenospermia compared with to controls (35). Moreover, the sperm flagella immunostained with acetylated α-tubulin were shorter in ADPKD patients. It suggested that the shortened flagella may contribute to poor sperm motility in ADPKD males.

**Figure 5:**
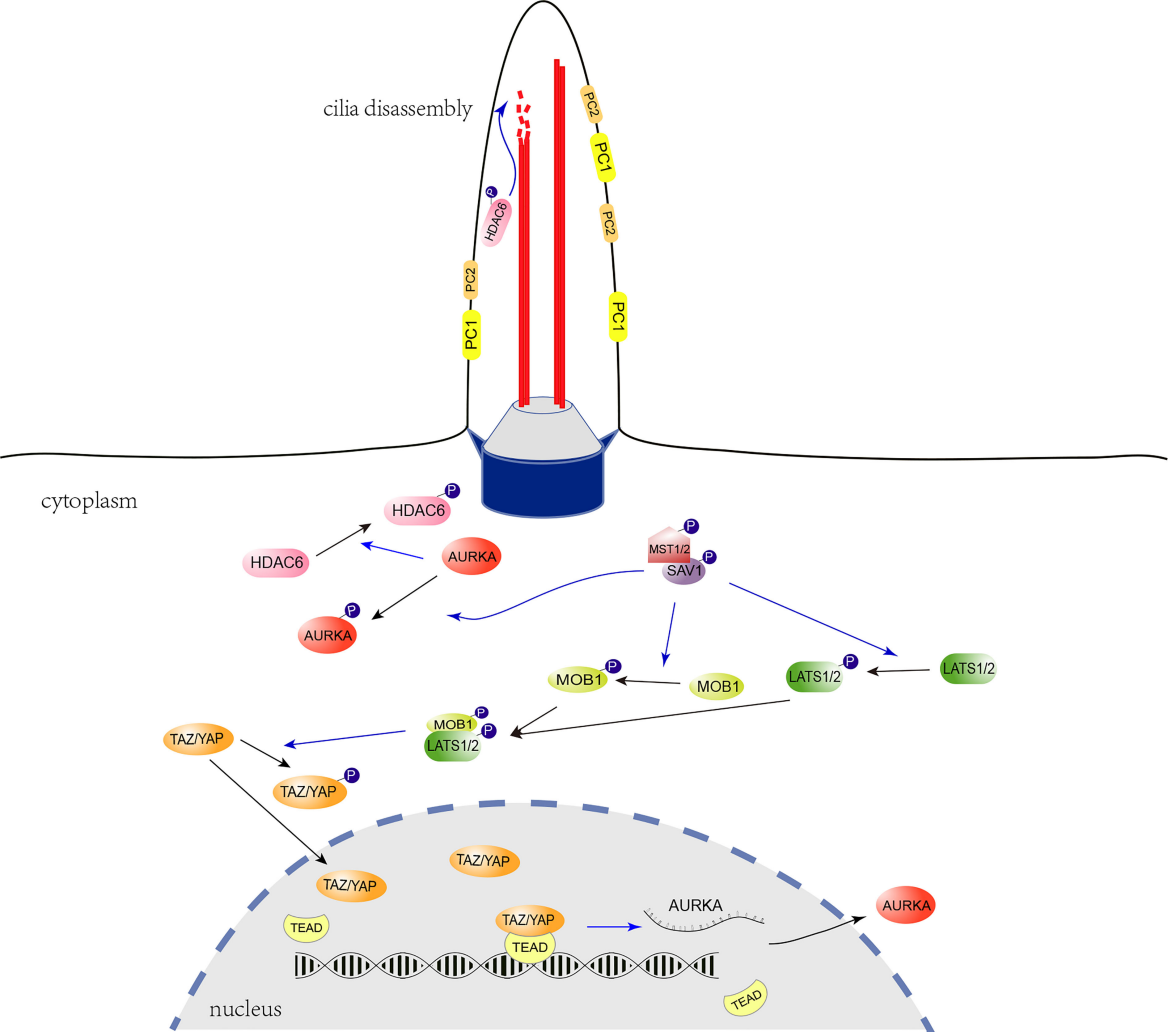
Regulatory role of the Hippo signaling pathway in ciliogenesis. The cilium is indicated with two microtubule doublets (red robs) bounded with the ciliary membrane, the transition zone (grey trapezoidal cylinder), and the basal body (blue cylinder) formed with the mother centriole. In the Hippo signaling pathway, Activated LATS1/2 phosphorylate and inhibit the translocation of YAP/TAZ from the cytoplasm to the nucleus. LATS1/2 is activated by MST1/2 through direct phosphorylation, which is promoted by the scaffold protein, phosphorylated SAV1. The YAP/TAZ act as transcriptional coactivators and interact with the TEAD transcription factor 4 (TEAD 4) to regulate the expression of *Aurka*. The AURKA phosphorylates and activates the tubulin deacetylase HDAC6 to promote ciliary disassembly. In the absence of PC1, decreased MST1 and LATS1 may promote YAP/TAZ nuclear translocation, facilitate *Aurka* transcription and subsequent ciliary disassembly. The blue-arrow lines indicate positive regulation.

The Hippo signaling pathway, which is highly conserved in mammals, is essential for controlling organ size and plays a vital role in cancer (36). Recently, it has been shown that YAP, one of the core components of the Hippo signaling, is associated with primary cilia growth (37–40). To investigate whether the Hippo signaling pathway is relevant to the pathogenesis of ADPKD, we detected YAP/TAZ expression in *Pkd1*-depleted mouse kidney tubule cells. It was found that phosphorylation level of YAP/TAZ was apparently reduced in *Pkd1*^-/-^ cells, which promoted the of YAP/TAZ translocation to the nucleus. Consequentially, proliferation of *Pkd1*^-/-^ cells was accelerated and the level of AURKA, a cilium disassembly-related protein, was obviously elevated under the regulation of nuclear YAP/TAZ. To further investigate the altered Hippo signaling, it was identified that MST1 and LATS1, the upstream kinases of YAP/TAZ, were both prominently decreased in the absence of PC1. Notably, it was reported that Hippo signaling pathway promotes ciliogenesis through preventing AURKA from forming the complex with the HDAC6 to stabilize the ciliary axoneme (41, 42). HDAC6 has been recognized to exacerbate cyst growth in ADPKD through enhancing cAMP signaling and upregulating epidermal growth factor receptor (EGFR) activity (43). As a result of reduced of MST1 and increased AURKA in the *Pkd1*-depleted cells, the function of HDAC6 would be enhanced, which would subsequently promote the ciliary disassembly (**Figure 5**).

Nevertheless, there are some limitations to this study. First, limited to ADPKD patients recruited from the reproductive outpatient department, it must be acknowledged that there was a selection bias and all of ADPKD subjects had abnormal semen parameters. We did not encounter ADPKD subjects without infertility as a control group to further verify the association of flagellar abnormalities with infertility. Although, it is difficult to conclude that all male patients with ADPKD are infertile, a certain proportion of infertility in male patients with ADPKD has been reported (7, 9, 44). Second, the structure of spermatozoa was not detected in all patients due to the limited sperm samples in 5 patients (Nos. 9-13). Thus, the flagellar abnormalities identified in this study may partially explain male infertility in ADPKD, but cannot account for the manifestations of infertility in all ADPKD patients, which warrants further investigation.

## 5 Conclusions

In conclusion, we highlighted the association between ADPKD and male infertility. Male ADPKD patients showed defects in the sperm morphology and shortened length of in sperm flagella. In the absence of PC1, MST1 and LATS1, the upstream components of the Hippo signaling pathway, were apparently reduced, which not only led to hyperactivation of YAP/TAZ, but also promoted AURKA/HDAC6-dependent ciliary disassembly. Our results demonstrated that the restrained Hippo signaling played a vital role in abnormal ciliogenesis and was potentially involved in the pathogenesis of flagellar defects in ADPKD.

## Data availability statement

The datasets presented in this study can be found in online repositories. The names of the repository/repositories and accession number(s) can be found in the article/**Supplementary Material**.

## Ethics statement

This study was prospectively approved by the ethics review committee of IPMCH. The patients/participants provided their written informed consent to participate in this study.

## Author contributions

C-MX and H-FH conceived the study and edited the paper. W-HS and Z-YZ carried out cell experiments and drafted the paper. M-JY, N-XQ, and Z-RJ collected semen samples and conducted sperm analysis. N-XX, X-YZ, X-LC, and S-CC analyzed the data. Z-YZ and M-JY made the figures. All authors contributed to the article and approved the submitted version.
