# Supplementary material for: Sperm morphological abnormalities in autosomal dominant polycystic kidney disease are associated with the Hippo signaling pathway via PC1
Source: Front Endocrinol (Lausanne). 2023 Apr 19;14:1130536. doi: 10.3389/fendo.2023.1130536 (PMC10155925; doi:10.3389/fendo.2023.1130536)
Supplement: Supplementary file 1 [file DataSheet_1.docx]

Supplementary Material

Sperm morphological abnormalities in autosomal dominant polycystic kidney disease are associated with the Hippo signaling pathway *via* PC1

Wei-Hui Shi^1#^, Zhi-Yang Zhou^1#^, Mu-Jin Ye^2^, Ning-Xin Qin^3^, Zi-Ru Jiang^1^, Xuan-You Zhou^1^, Nai-Xin Xu^2^, Xian-Lin Cao^1^, Song-Chang Chen^1^, He-Feng Huang^1,2,4*^, Chen-Ming Xu^1, 2*^

^1^Obstetrics and Gynecology Hospital, Institute of Reproduction and Development, Fudan University, Shanghai, P. R. China

^2^International Peace Maternity and Child Health Hospital, Shanghai Jiao Tong University School of Medicine, Shanghai, P. R. China

^3^Department of Assisted Reproductive Medicine, Shanghai First Maternity and Infant Hospital, Tongji University School of Medicine, Shanghai, P. R. China

^4^Research Units of Embryo Original Diseases, Chinese Academy of Medical Sciences (No. 2019RU056), Shanghai, China.

***Correspondence:**Chen-Ming Xu (chenming_xu2006@163.com, 419 Fangxie Road, Shanghai, P. R. China); He-Feng Huang (huanghefg@hotmail.com, 419 Fangxie Road, Shanghai, P. R. China)

# These authors contributed equally to this study.

# Supplementary Figure


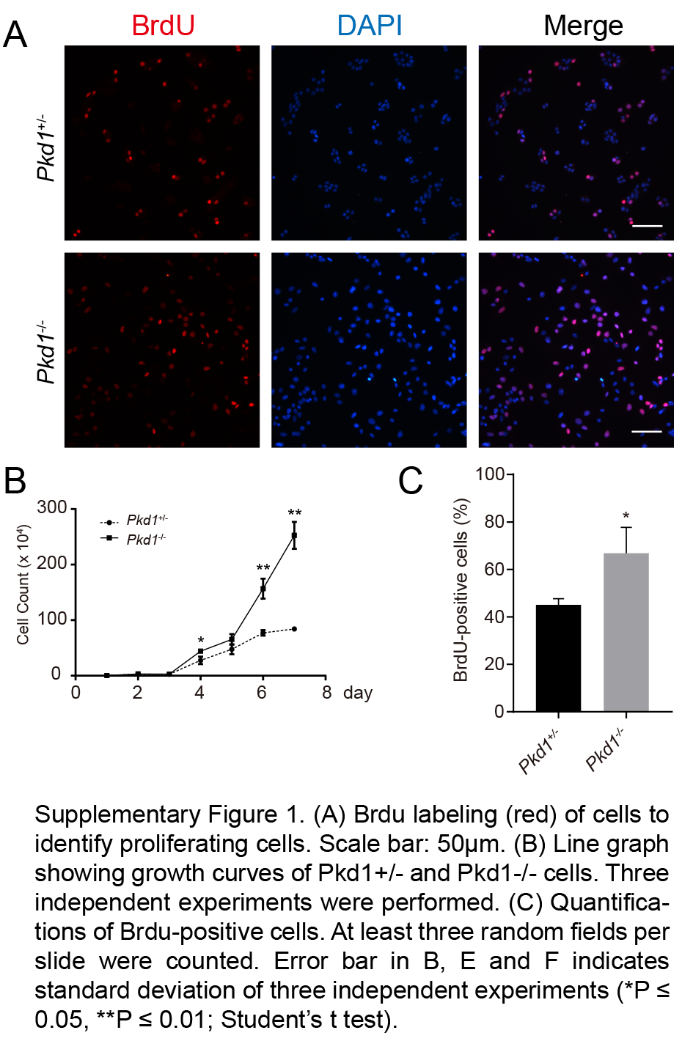


**Supplementary Figure 1.** (A) Brdu labeling (red) of cells to identify proliferating cells. Scale bar: 50μm. (B) Growth curves of *Pkd1*+/- and *Pkd1*-/- cells. Three independent experiments were performed. (C) Quantification of Brdu-positive cells. At least three random fields per slide were counted. Error bar in B and C indicates standard deviation of three independent experiments (**P* ≤ 0.05, ***P* ≤ 0.01)
